# Host–Virus Interface in Persistent SARS-CoV-2 Infections: Viral Characteristic Evolution and Gene Expression Profiling Analysis

**DOI:** 10.3390/ijms26136221

**Published:** 2025-06-27

**Authors:** Athok Shofiudin Maarif, Yukari Nishikawa, Miyako Takata, Kyosuke Kanai, Edo Riyandani, Kengo Mukuda, Momone Mimura, Kosuke Yamaguchi, Hiroyuki Kato, Ryo Okamoto, Kensaku Okada, Tsuyoshi Kitaura, Masaki Nakamoto, Akira Yamasaki, Seiji Kageyama, Hiroki Chikumi

**Affiliations:** 1 Division of Infectious Diseases, Graduate School of Medicine, Faculty of Medicine, Tottori University, Nishi-cho 86, Yonago 683-8503, Tottori, Japan; athok.arief@yahoo.co.id (A.S.M.); nyukari.dn.nh@gmail.com (Y.N.); riyandani.edo@gmail.com (E.R.); kengom02@gmail.com (K.M.); katuketu525@gmail.com (H.K.); b11m1021t@tottori-u.ac.jp (R.O.); kensaku-o@tottori-u.ac.jp (K.O.); kitaurat@tottori-u.ac.jp (T.K.); nakamoto@tottori-u.ac.jp (M.N.); 2 Division of School of Health Science, Department of Pathobiological Science and Technology, Faculty of Medicine, Tottori University, Yonago Nishi-cho 86, Yonago 683-8503, Tottori, Japan; miyakot@tottori-u.ac.jp (M.T.); quarmm0504@gmail.com (M.M.); 3 Division of Virology, Department of Microbiology and Immunology, Faculty of Medicine, Tottori University, Nishi-cho 86, Yonago 683-8503, Tottori, Japan; kkanai@tottori-u.ac.jp (K.K.); skageyama@tottori-u.ac.jp (S.K.); 4 Department of Multidisciplinary Internal Medicine, Division of Respiratory Medicine and Rheumatology, Faculty of Medicine, Tottori University, Nishi-cho 86, Yonago 683-8503, Tottori, Japan; kske2@tottori-u.ac.jp (K.Y.); yamasaki@tottori-u.ac.jp (A.Y.)

**Keywords:** SARS-CoV-2, persistent infection, host–virus interaction, gene expression, GO enrichment, immune response, viral evolution

## Abstract

Persistent SARS-CoV-2 infections involve prolonged viral replication and immune system interactions, potentially driving viral evolution and immune escape. This study examines viral characteristics and host gene expression changes in persistent infections. The nasopharyngeal samples from four patients with persistent SARS-CoV-2 infections at Tottori University Hospital, Japan, were analyzed. Viral isolates were cultured, and infectivity was assessed using TCID_50_ assays. To investigate host responses, RNA sequencing (RNA-seq) was performed to identify differentially expressed genes (DEGs), and Gene Ontology (GO) enrichment analysis mapped affected biological pathways. Viral genome sequencing detected mutations associated with prolonged infection. The results showed significant infectivity differences between early- and late-phase infection. Gene expression analysis revealed a strong early phase of pro-inflammatory response (IL6, TNF, IL1B, CXCL10) followed by immune suppression. GO enrichment analysis highlighted inflammation and cytokine-mediated immune pathways. Genomic sequencing identified mutations in ORF1ab and the spike (S) protein, potentially aiding immune escape. The findings underscore that SARS-CoV-2 adapts during persistent infections, altering infectivity and immune responses. These highlight the need for continued monitoring of prolonged infections to mitigate immune escape and viral evolution.

## 1. Introduction

The coronavirus disease 2019 (COVID-19), which is caused by severe acute respiratory syndrome coronavirus 2 (SARS-CoV-2), has had a continuous impact on global health since 2019 [1,2,3]. Patients infected with SARS-CoV-2 may experience a wide range of clinical outcomes, from mild to moderate disease with recovery to severe respiratory failure, which leads to death [4]. In some cases, infection with SARS-CoV-2 can develop over an extended period, which is known as persistent infection [5,6]. However, the exploration of persistent SARS-CoV-2 infection is still understudied, yet some individuals have experienced prolonged and persistent infection, which has become an increasing issue. This phenomenon has raised concerns about the potentially profound and lasting impact on long-term public health and the management of the COVID-19 pandemic, underscoring the urgent need for further research in this area [7,8].

Persistent SARS-CoV-2 infection is characterized by the prolonged presence of the virus in an individual, often due to an inability to clear the infection effectively [9]. In most cases, the SARS-CoV-2 virus is no longer found in the respiratory tract after the acute phase of infection [10]. A comprehensive review found that the infectious virus is typically cleared from respiratory secretions within 9–14 days of symptoms starting, while viral RNA can still be detected within 30 days, regardless of vaccination status or the virus variant [6]. According to later studies, some cases can lead to viral persistence, which was confirmed through the serial isolation of infectious viruses for extended periods of viral shedding during a persistent infection that can last for at least 30 days or more; this also may further suggest immune escape variants [11,12].

This persistent condition is where the virus can continue replicating and mutating over extended periods [13]. Understanding the mechanisms of viral persistence, the evolution of the resulting viral–host interaction, and the clinical implications is essential for developing effective management and treatment strategies [14]. Continuous research and vigilant monitoring are crucial to addressing the complexities associated with these persistent infections.

In this research study, we investigated changes in viral properties and virus–host cell response patterns through a comprehensive analysis and examination of transcriptome profiles. These findings offer a new understanding of the development and progression of COVID-19 caused by SARS-CoV-2 and suggest novel approaches for the prevention and control of the spread of SARS-CoV-2.

## 2. Results

### 2.1. Clinical Patient Data and the Characteristics of SARS-CoV-2 Isolates

We collected persistent SARS-CoV-2 infection samples from four clinical patients during a sampling period in Tottori University Hospital, Japan (Table 1). The persistent or prolonged duration of infection ranged from 37 to 58 days. The mean age of the patients was 64.5 years (±19.16 years). All patients had underlying conditions and comorbidities such as cancer, rheumatoid arthritis, and hypertension, and one patient was pregnant. Three patients received antiviral treatment with remdesivir (*n* = 2) or molnupiravir (*n* = 1), and one patient did not receive any medicine due to pregnancy.

**Figure 1:**
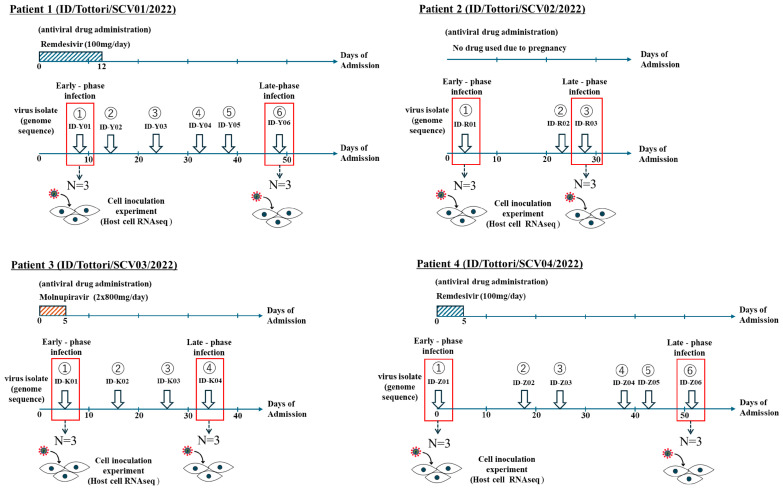
Patient sampling chart in persistent infection with SARS-CoV-2. Samples were collected from four clinical patients (ID/Tottori/SCV01/2022, ID/Tottori/SCV02/2022, ID/Tottori/SCV03/2022, ID/Tottori/SCV04/2022) with a total of 19 SARS-CoV-2 samples that fulfilled the inclusion criteria. The red box indicates the samples that were categorized as early-phase infection and late-phase infection.

**Figure 2:**
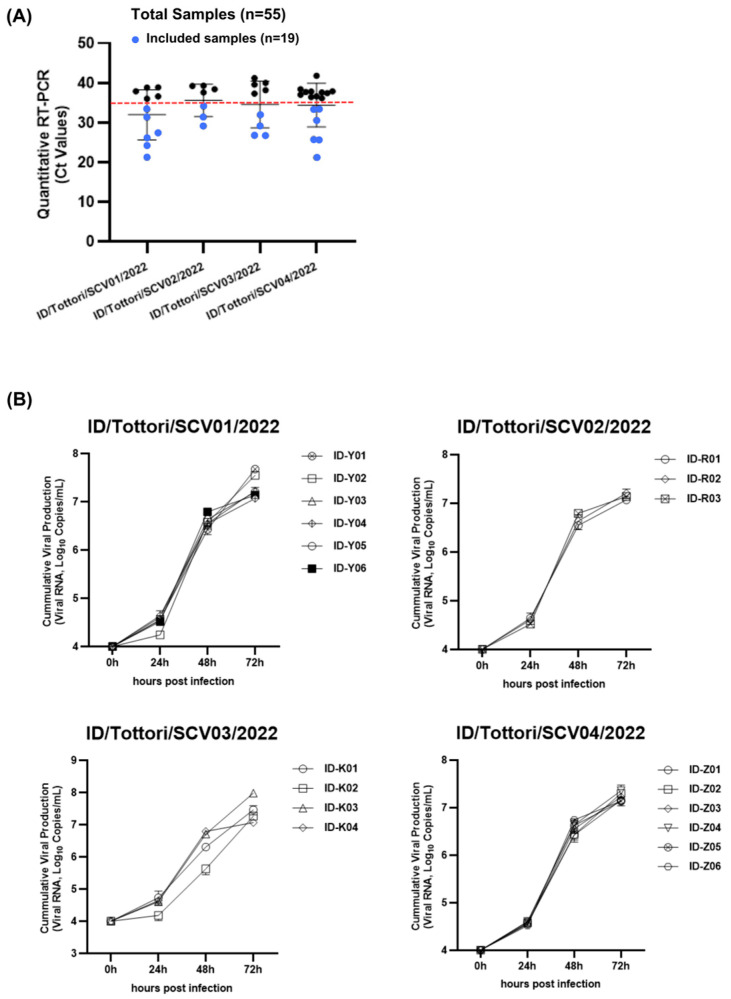
The growth kinetics of SARS-CoV-2 virus isolates. (**A**) From the 55 clinical samples, 19 samples (blue dot) with a Ct value of ≤35 were included as the experimental samples. The red dotted line represents the Ct value of 35. (**B**) The in vitro viral growth kinetics in the culture are shown for 19 SARS-CoV-2 isolates from 4 clinical patients. (ID/Tottori/SCV01/2022, ID/Tottori/SCV02/2022, ID/Tottori/SCV03/2022, ID/Tottori/SCV04/2022).

We collected 55 clinical nasopharyngeal samples with cycle threshold (Ct) values ranging from 21.2 to 40; these were identified using quantitative real-time PCR (RT-qPCR). Then, 19 samples from four patients (Figure 1) that had Ct values of ≤35 (Figure 2A) were selected for viral inoculation to perform further analysis in this experiment. We successfully inoculated all 19 samples (Ct ≤ 35) in VERO-E6/TMPRSS2 cells.

Then, viral growth kinetics were assessed at four different time points (0, 24, 48, and 72 h) using a primary culture system with VERO-E6/TMPRSS2 cells. After the inoculation of the virus (10^4^ SARS-CoV-2 RNA copies), the virus showed stable growth activity in a range of 1.1 × 10^7^–3.9 × 10^7^ copies/mL in 72 h (Figure 2B). The growth kinetics of the virus from all patients showed no significant changes between the early and late phases of persistent infection.

### 2.2. Dynamic Changes in Infectivity Across Persistent COVID-19 Infection

Viral infectivity was assessed using the Tissue Culture of Infectious Dose (TCID_50_) assay with VERO-E6/TMPRSS2 cells. After the virus (10^4^ SARS-CoV-2-RNA copies) was inoculated for 72 h, the TCID_50_ was calculated throughout the infection.

**Figure 3:**
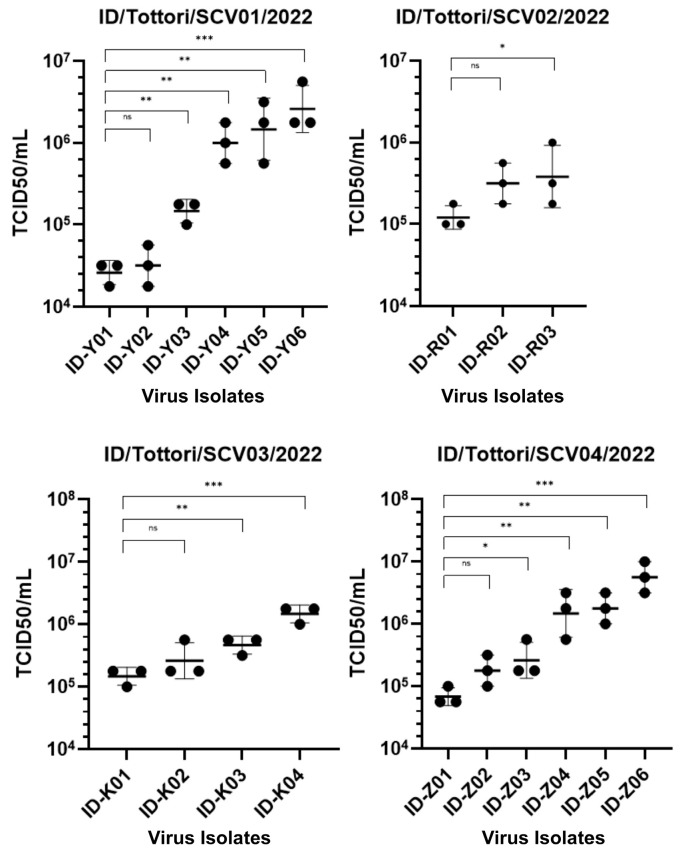
TCID_50_ assay showing the infectivity of 19 SARS-CoV-2 isolates from four clinical patients (ID/Tottori/SCV01/2022, ID/Tottori/SCV02/2022, ID/Tottori/SCV03/2022, ID/Tottori/SCV04/2022). The X-axis represents different virus isolate IDs. The Y-axis represents the infectivity titer is expressed as TCID_50_/mL from the biological triplicate of all viral isolates. Lower TCID_50_ values indicate higher infectivity, indicating a greater capacity of the virus to infect cultured cells at lower doses. Statistical significance is tested using a *t*-test. The error bars indicate the mean ± SD. (ns) means not significant, (*) indicates a significant difference, * *p* < 0.05, ** *p* < 0.01, *** *p* < 0.001.

Interestingly, there were increasing changes in the infectious viral titer (TCID_50_) (Figure 3). During the persistent infection, the virus showed significant changes in infectivity in all patients. We found that in the early-phase infection, the virus had a lower TCID_50_ value, and it increased throughout the late-phase infection. This means that in the early-phase infection, the virus had high infectivity, which then decreased during the late-phase infection.

### 2.3. Comparison of Inflammatory Gene Expression Patterns in Host Cells

**Figure 4:**
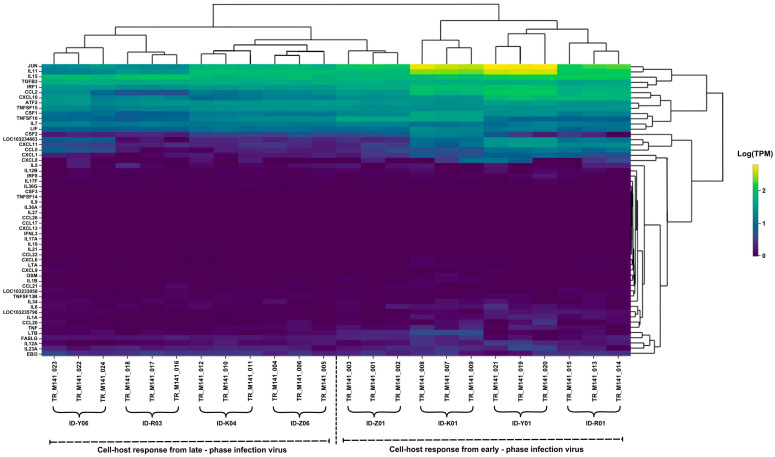
Expression patterns of the host cytokine genes from the RNA-sequencing analysis of the SARS-CoV-2 isolates.

We performed a comparative transcriptome analysis to further investigate the host response against the persistent infection and virus–host cell immune interaction in SARS-CoV-2 infection. We used sets of antiviral capacity and inflammation response genes [15] to quantify inflammatory gene expression patterns during persistent infection. For this purpose, VERO-E6/TMPRSS2 cells were infected with the early-phase virus and late-phase virus (in vitro), and the expressions of cytokine genes were compared using RNA-seq. The heatmap analysis from RNA-sequencing data (Figure 4) reveals significant changes in gene expression during SARS-CoV-2 infection, highlighting distinct immune responses in the early and late phases of infection.

For the cell host response in the early-phase infection, genes such as JUN, IL15, ATF2, TNFSF15, and CXCL10 were significantly upregulated, reflecting a strong pro-inflammatory and immune response. TNF (tumor necrosis factor), IL6, and IL1B, which are major pro-inflammatory cytokines, were also highly expressed in this phase.

During the late-phase infection, the cell host response showed that most cytokines and chemokines were notably decreased in expression, indicating a decline in immune cell recruitment. However, although this was reduced, certain pro-inflammatory genes, including JUN, IL15, ATF2, TNF, IL6, and IL1B, continued to be expressed.

### 2.4. Gene Ontology (GO) Enrichment Analysis of DEGs

**Figure 5:**
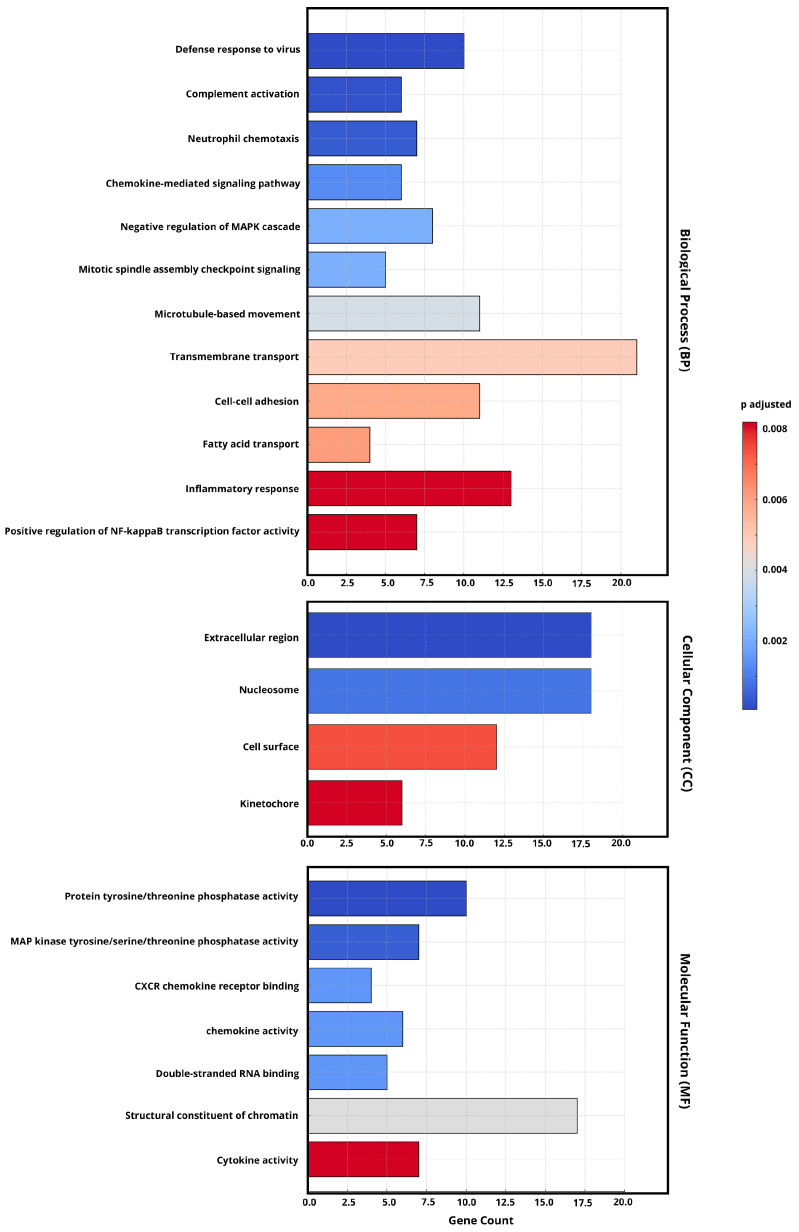
The GO enrichment analysis of DEGs from the early and late phases of persistent SARS-CoV-2 infection (FDR < 0.05, |log_2_FC| ≥ 1). The most significantly enriched gene pathways (*p*-values (Padj) < 0.05) were selected and then categorized into the biological process (BP), cellular component (CC), and molecular function (MF) categories. The bar color indicates adjusted *p*-values (Padj), with darker red representing higher statistical significance. The x-axis represents the gene count of DEGs associated with each GO term.

Next, we analyzed the differential pathways among the viruses in the persistent infection in detail by conducting a Gene Ontology (GO) enrichment analysis (Figure 5) based on their respective differentially expressed genes (DEGs). This showed that, compared with the late-phase infection, the upregulated DEGs in necessary biological process (BP) pathways in the early-phase infection were mainly enriched in inflammation-related pathways, including inflammatory response and positive regulation of nuclear factor kappa B (NF-kB) transcription factor activity.

From the perspective of cellular components (CCs), the enrichment of cell surface and kinetochore terms suggests that viral interactions with host membranes remain significant in prolonged infection. In addition, the molecular function (MF) analysis highlights highly expressed cytokine activity.

### 2.5. Gene Expression Profiling of Human Host Cell Response

To further investigate the possible pattern of viral–host cell response capacity and cytokine production activities during persistent infection with SARS-CoV-2, we studied the interaction of the persistent virus with the Calu-3 human airway epithelial cell line (in vitro) as a human host cell model for SARS-CoV-2 infection.

**Figure 6:**
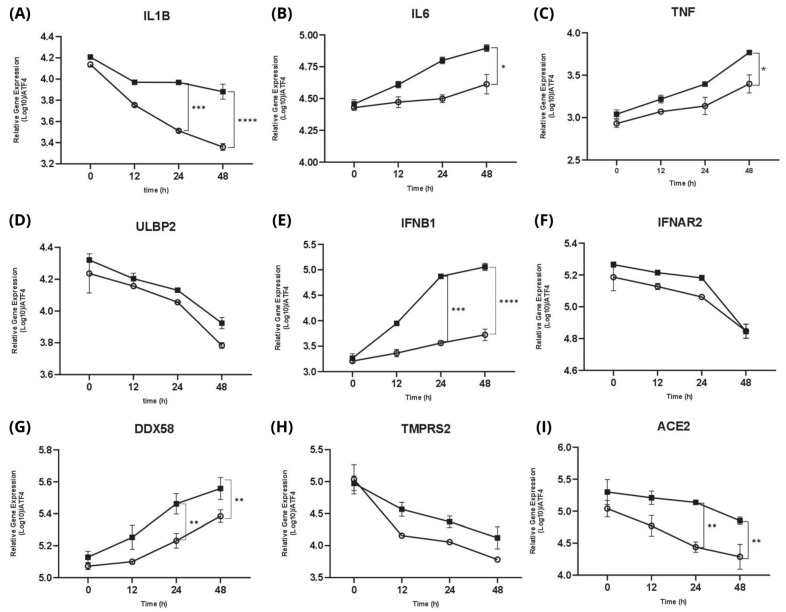
Quantitative real-time PCR (RT-qPCR) summarizing the gene expression of all isolates in early-phase and late-phase infection. Gene expression levels were measured at 0, 12, 24, and 48 h post-infection. ■ represents the early-phase infection, and ○ represents the late-phase infection. (**A**) IL1B expression significantly declines from early to late phase infction. (**B**) IL6 and (**C**) TNF showes gradually increasing expression of pro-inflammatory cytokines compared from early to late phase infection (**D**) ULBP2 expression demonstrates decrease expression over time. (**E**) IFNB1 markedly induced a gradually increased expression compared from early to late phase infection. (**F**) IFNAR2 remains slight downward expression trend. (**G**) DDX58 increasing more prominently in the early phase, particularly at later time points. (**H**) TMPRSS2 and (**I**) ACE2, both as the cell entry genes demonstrate expression across both phases, especially ACE2 shows significantly downregulated over time compared from early to late phase infection. Statistical significance is tested using a *t*-test. The error bars indicate the mean ± SD. (*) indicates a significant difference, * *p* < 0.05, ** *p* < 0.01, *** *p* < 0.001, and **** *p* < 0.0001.

We performed time-course experiments to investigate the significant changes in transcripts in response to SARS-CoV-2 infection. Infected groups were analyzed at four specific time points: 0, 12, 24, and 48 h post-infection (hpi). We evaluated three types of genes: cell regulator genes involved in the inflammatory response, the induction of antiviral-related genes, and interferon production; pathogen recognition receptors (PRRs) for initiating innate immune responses; and virus receptors for cell entry. Interestingly, we found that the virus–host cell response for nine genes exhibited decreased expression of gene expression from the early-phase to the late-phase infection (Figure 6).

For each gene, among the inflammatory cytokines, IL6 and TNF (Figure 6B,C) indicate the gradually increasing expression of pro-inflammatory cytokines. In addition, ULBP2 (Figure 6D) was highly expressed at 0 hpi and then gradually decreased. Then, we observed the gradually increased expression of IFNB1 (Figure 6E). DDX58, also known as RIG-I (retinoic acid-inducible gene I), was upregulated (Figure 6G).

Lastly, for the cell entry genes ACE2 and TMPRSS2 (as the receptor of the SARS-CoV-2 cell entry mechanism), we observed a significant decrease in expression; they were highly expressed during early infection (0 hpi) and constantly decreased during the late-phase infection (Figure 6H,I).

### 2.6. Distinct Genomic Analysis Between Early-Phase and Late-Phase Infection

**Figure 7:**
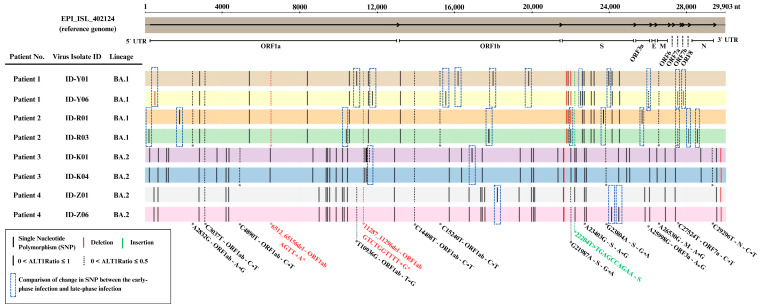
Genomic sequence analysis of the SARS-CoV-2 virus between early-phase and late-phase infection.

Finally, to enhance the investigation of the genomic analysis pattern, we aligned sequences to the SARS-CoV-2 reference genome from the GISAID database (EPI_ISL_402124). We analyzed viral genomic variations between the early-phase (ID-Y01, ID-R01, ID-K01, ID-Z01) and late-phase (ID-Y06, ID-R03, ID-K04, ID-Z06) infection groups, identifying single-nucleotide polymorphisms (SNPs), insertions, and deletions in key genomic regions, including the ORF1ab, S, ORF3a, M, and N genes (Figure 7).

Our analysis revealed that genetic changes, including single-nucleotide polymorphisms (SNPs), insertions, and deletions, were more commonly observed in the genomic regions of the ORF1ab region (13 SNPs, 1 Deletion) and spike (S) region (5 SNPs, 1 deletion) when comparing the early-phase and late-phase SARS-CoV-2 genomes (Table 2).

## 3. Discussion

This study provides insights into the interactions between host immunology responses and viral behavior during persistent SARS-CoV-2 infection, revealing biological behaviors in comparison with acute infection. While most prior research has centered on acute phases of COVID-19, our study offers a comprehensive analysis of persistent cases by combining virological assays, gene expression profiling, and viral genome analysis. To summarize, we observed a marked decrease in viral infectivity from early- to late-phase samples, alongside a sustained and dysregulated inflammatory response in host cells. Genomic analysis revealed common mutations, particularly in the ORF1ab and spike (S) regions. Interestingly, despite there being no antiviral pressure, viral evolution and immune escape patterns were still evident, suggesting that natural selection plays a critical role. These integrated findings highlight persistent infection as an active and evolving process that may contribute to prolonged symptoms, viral adaptation, and possibly the emergence of immune-escape variants.

One of the key observations in this study is the discrepancy between viral replication kinetics and infectivity during persistent infection. The results of the TCID_50_ assay (Figure 3) showed early-phase isolates had lower TCID_50_ values than late-phase, indicating higher infectivity in the early-phase infection and which declined in late-phase infection, while the viral replication kinetics remained relatively stable (Figure 2B). This shift in viral behavior has been reported in the global transition from Delta to Omicron, where Omicron exhibited reduced replication in lung epithelial cells and lower pathogenicity but increased transmissibility and immune escape [16,17]. However, previous studies on intra-host interactions [18,19] have mainly focused on genomic changes of the virus but have lacked the biological behavior of the virus. Furthermore, many previous studies have focused on cross-sectional comparisons such as between healthy individuals and COVID-19 patients, or single-timepoint snapshots of viral genomic evolution, while longitudinal analyses tracking temporal changes within the same group remain insufficiently reported. This study offers a longitudinal analysis by tracking early- and late-phase infection within the persistent infection patients’ cohort. Therefore, our study brings new insight into this field which explores both aspects of genetic and biological characteristic changes during intra-host evolution during the persistent SARS-CoV-2 infection.

The host immune response analysis revealed important findings related to the inflammatory responses observed in host cells during persistent infection. Host transcriptomes corresponding with the host cell responses were compared (Figure 4), especially in the inflammatory process. During early-phase infection, there was robust upregulation of pro-inflammatory cytokines such as JUN, IL15, ATF2, TNFSF15, and CXCL10, indicating strong immune activation. These cytokines and chemokines collectively orchestrate the initial immune response to SARS-CoV-2 by promoting inflammation, immune cell recruitment, and antiviral signaling [20]. Furthermore, significant upregulation of IL6, TNF, and IL1B in the early phase of SARS-CoV-2 infection is crucial and a key factor for activating an effective immune response in the SARS-CoV-2 infection process [21]. In contrast, the late phase was characterized by altered expression of TNF and IFNAR2; in addition, the continued expression of IL6 and IL1B in the late phase indicates a persistent inflammatory state. This ongoing expression suggests a persistent inflammatory response that is often observed in prolonged infections. Lucas et al. [22] and Blanco-Melo et al. [23] stated that this prolongation of the inflammatory response suggests a dysregulated immune state, potentially contributing to sustained pathology and chronic symptoms. Notably, immune suppression in patients can significantly alter cytokine profiles, leading to impaired antiviral responses. Studies [24,25] have shown that dysregulated proinflammatory cytokines such as IFNs, IL6, and TNF may compromise viral clearance while minimizing tissue damage, creating a condition that supports persistent infection despite low infectivity. These imbalances in cytokine expression are frequently implicated in cytokine storms and severe disease outcomes in COVID-19 patients.

To further explore this viral adaptation to the host cell responses, our analysis found significant upregulation of the enrichment of genes involved in inflammatory pathways, including inflammatory response and the positive regulation of NF-κB activity (Figure 5). This pathway regulates immune responses and cytokine production, both of which remain highly active in prolonged infections [26]. In addition, the molecular function (MF) analysis highlights highly expressed cytokine activity. which is crucial in coordinating the immune response [27]. Previous studies [28,29] also emphasized that these pathways are commonly activated in persistent viral infections and may indicate ongoing immune dysregulation or viral escape through immune signaling pathways. These observations were further validated by using Calu-3 cells infected with persistent SARS-CoV-2 isolates, which triggered strong induction of IL6, TNF, and IFNB1 (Figure 6B,C,E). These cytokines play a crucial role in the inflammatory response in virtually all infectious diseases and are produced by various cell types [30]. DDX58 (Figure 6G) was also highly expressed which, as expected, implies the critical role of signaling in evading the host immune response and may contribute to the virus’s ability to establish infection [31]. ACE2 and TMPRSS2 (Figure 6H,I) were decreasingly expressed. These, as a result of interferon-mediated immune responses, act to limit viral spread by modulating host gene expression, including the downregulation of ACE2 and TMPRSS2 [32]. Recent studies showed that the gradual increase in the pro-inflammatory cytokines IL6, TNF, and IFNs underscores their pivotal roles in mediating the inflammatory response during SARS-CoV-2 infection [28,33,34]. Our data support the view that persistent infection maintains an inflammatory environment, possibly promoting immune escape and long-term host pathology.

Next, our genomic analysis further revealed that persistent infection is accompanied by commonly observed mutations, particularly in the ORF1ab and spike (S) regions of the SARS-CoV-2 genome. Genomic mutations in ORF1ab, which is known to influence viral RNA synthesis, replication, and translation, may reflect adaptations that enhance viral fitness or help evade host defenses [35]. Meanwhile, SNPs in the spike region, which is under strong selective pressure due to its role in cell entry and infectivity, could contribute to variations in disease severity [36]. Interestingly, the number of SNP changes between early- and late-phase viruses was greater in BA.1 compared to the BA.2 sub-lineage. A previous study showed that the BA.1 lineage has more comparative mutations among the other omicron sub-variants such as BA1.1., BA.2, and BA.3 [37]. This may reflect differences in intra-host mutational dynamics between Omicron sub-lineages, potentially influenced by viral replication, immune escape pressure, or host-specific factors. This supports the idea that persistent infections create an environment where the virus can gradually evolve, potentially generating variants with altered fitness or pathogenicity in persistent infection.

Interestingly, we found that viral evolution was observed even in the absence of antiviral treatment. Some previous studies have investigated how antiviral treatments such as remdesivir could drive mutations that are resistant to or enhance the replication efficiency of the virus [11,38,39]. However, our study included a sample of patients who did not receive any antiviral treatments, but such viral evolution and adaptation were still exhibited. Persistent infections could create environments conducive to viral adaptation and the emergence of variants capable of immune escape [11,13]. This result might indicate that natural selection, shaped by host immune responses and intrinsic viral factors, is equally significant in driving viral evolution and immune escape mechanisms.

Nonetheless, this study has several limitations. The first is regarding reliance on Vero-E6/TMPRSS2 cells for transcriptome profiling. These cells are known to be IFN-deficient and may not fully replicate the host immune environment in human airway epithelium [40]. Although we partially addressed this by validating cytokine responses in Calu-3 cells, future prospective studies using primary human airway cells or organoid systems will be important to fully capture the interactions between persistent SARS-CoV-2 and the host, and are warranted to validate our findings. Second, this study used N2-targeted RT-qPCR, which reflects total viral RNA but does not distinguish between genomic (gRNA) and subgenomic mRNA (sgmRNA). Future studies should apply strand-specific or specifically RdRp-targeted assays to better assess viral replication in persistent infection. Third, as a single-center study with a limited sample size, our findings may not fully represent broader population dynamics. Additionally, although patients did not receive standardized antiviral therapy, sex, age, and certain comorbidities such as cancer or pregnancy, as well as treatment regimens, were not evenly distributed between the groups, which may have influenced immune responses and viral evolution. To address this, we analyzed overall patterns and trends, such as changes in viral characteristics and immune response assays across the patient cohort. This approach allows us to provide meaningful insights and identify consistent findings despite the variability in individual conditions. These findings, although preliminary, provide a meaningful foundation for future multi-center studies with larger cohorts to further clarify the mechanisms underlying SARS-CoV-2 persistence.

This study highlights how the SARS-CoV-2 virus can evolve and persist within the host by gradually adapting to immune pressures. Higher viral infectivity in the early-phase of infection corresponds with significant upregulation of key proinflammatory cytokine to accommodate the virus replication and entry. These changes reflect a unique viral strategy to survive long-term within the host. By combining viral and host cell genomic changes between the early and late phase of infection, our findings offer new insights into how persistent viruses unfold and how the evolution of immune escape might lead to persistent infection. Understanding this process is crucial as we prepare for the long-term impact of COVID-19.

## 4. Materials and Methods

### 4.1. Clinical Sample Collection

This study used a total sampling approach, including all patients diagnosed with persistent SARS-CoV-2 infection during the defined sampling period from 1 January 2022, until 31 December 2022, at Tottori University Hospital, Japan. There were 4 patients diagnosed with persistent prolonged COVID-19. From these patients, we collected a total of 55 nasopharyngeal specimen samples. However, in our study, we only included samples with a quantitative RT-PCR cycle threshold (Ct) value of ≤35, as previous studies have indicated that this threshold ensures a sufficient viral load for accurate and reliable results of viral inoculation [9,41,42]. Therefore, 19 samples met this criterion, were prepared for viral inoculation, and were used in our experiment. Then the samples were selected for the early-phase infection and late-phase infection.

The early-phase infection was defined as the period nearest to the hospital admission date, when patients had the lowest Ct values and active infection was first confirmed. The late-phase infection was defined as the period nearest to the discharge date, when the Ct values were higher, and the patient’s condition had generally stabilized. This approach reflected the clinical trajectory of infection in persistent cases and allowed consistent classification across all patients.

### 4.2. SARS-CoV-2 Isolates Using VERO-E6/TMPRSS2 Cells

VERO-E6/TMPRSS2 cells were kindly obtained from Prof. Seiji Kageyama from the Division of Virology, Department of Microbiology and Immunology, Faculty of Medicine, Tottori University. The cells were cultured in Dulbecco’s Modified Eagle’s Medium (DMEM; FUJIFILM Wako, Osaka, Japan) supplemented with 5% fetal bovine serum (FBS) and 100 U/mL penicillin–streptomycin at 37 °C in a humidified atmosphere with 5% CO_2_. Nasopharyngeal specimens were collected from patients at Tottori University Hospital, Tottori University, Japan. The persistently positive samples were preserved in a viral transport medium (VTM; universal transport medium, COPAN Diagnostic Inc., Murrieta, CA, USA). All experiments on infectious viruses were performed in a biosafety level 3 (BSL3) laboratory at the Virology Department of Tottori University, Japan. Initially, the samples were filtered with a sterile 0.45 µM Minisart syringe filter. The complete confluent VERO-E6/TMPRSS2 cells (10^5^) were washed with phosphate-buffered saline (PBS) and inoculated with the filtered samples. After 1 h of inoculation, the cells were washed twice with PBS and maintained in 5%FBS-DMEM at 37 °C in a humidified atmosphere of 5% CO_2_. After 72 h, cells were observed to evaluate the cytopathic effect. The culture supernatants were subjected to make virus aliquots (500 µL) and extract the viral RNA to check the viral loads.

### 4.3. Calu-3 Human Airway Epithelial Cell Infection

Calu-3 cells (human airway epithelial cell line as a human host cell model for SARS-CoV-2 infection) [43] were purchased from the American Type Culture Collection (ATCC, Manassas, VA, USA). The cells were cultured in Eagle’s Minimum Essential Media (EMEM; FUJIFILM Wako, Osaka, Japan) supplemented with 10% fetal bovine serum (FBS), 500 µL L-glutamine, and 100 U/mL penicillin–streptomycin in 6-well plates at 37 °C in a humidified atmosphere with 5% CO_2_. Cells were washed with fresh EMEM and inoculated with 10^4^ viral copies of SARS-CoV-2 viruses of each strain in triplicate for 2 h at 37 °C in a humidified atmosphere with 5% CO_2_. After that, cells were washed three times with EMEM, followed by adding fresh medium to indicate 0 h of the experiment. The biological triplicate samples of both mock-infected and virus-infected Calu-3 cells were collected at various time points of 0,12, 24, and 48 h post-infection (hpi) for the gene expression assay. RNA isolation from Calu-3 cells was carried out using the RNeasy Mini Kit reagent (QIAGEN, Tokyo, Japan) according to the manufacturer’s instructions.

### 4.4. Assessment of Viral Growth Kinetics

VERO-E6/TMPRSS2 cells were cultured in DMEM supplemented with 5% fetal bovine serum (FBS) and 100 U/mL penicillin–streptomycin in 24-well plates at 37 °C in a humidified atmosphere with 5% CO_2_. The completed confluent cells were inoculated with viruses at a multiplicity of infection (MOI) of 5 in triplicate for 1 h at 37 °C/5%CO_2_. After that, cells were washed three times with 1xPBS, followed by the addition of fresh medium. Subsequently, the culture supernatants collected on days 1, 2, and 3 underwent RNA extraction and quantitative real-time PCR (qPCR) to establish growth curves for the representative SARS-CoV-2 strain isolates.

### 4.5. RNA Extraction and Viral Quantification qPCR

SARS-CoV-2 RNA in the culture supernatant (140 µL) was extracted and purified using a QiAmp Viral RNA Mini Kit (QIAGEN, Tokyo, Japan) according to the manufacturer’s protocol. We quantified the viral RNA using conventional real-time RT-PCR for SARS-CoV-2 while following the protocol established by the National Institute of Infectious Diseases in Japan [44]. N2 gene RNA (Nihon Gene Research Laboratories, Inc., Sendai, Japan) was diluted ten-fold to create a standard curve. Quantitative real-time PCR (qPCR) was conducted on a QuantStudio 5 system (Thermo Fisher Scientific, Waltham, MA, USA). The 20 µL reaction mixture for real-time RT-PCR included 5 µL of TaqMan Fast Virus 1-Step Master Mix (Thermo Fisher Scientific), 1.8 µL of forward primer, 1.8 µL of reverse primer, 0.5 µL of probe, 5.9 µL of RNAse free water, and 5 µL of the sample RNA. The primers and probes (Table 3) were specific to the N2 gene (Nihon Gene Research Laboratories, Inc.). The amplification conditions were an initial reverse transcription at 50 °C for 5 min and initial denaturation at 95 °C for 20 s, followed by 45 cycles at 95 °C for 15 s and 60 °C for 1 min.

### 4.6. Viral Infectivity Assessment

The viral infectivity assessment used the 50% tissue culture infectious dose (TCID_50_) per mL for the SARS-CoV-2 virus in VERO-E6/TMPRSS2 cells. The TCID_50_ assay determines the point at which 50% of cells in the culture are infected and provides the approximate viral titer [45,46]. The VERO-E6/TMPRSS2 cells (10^4^ cells/well) were seeded into 96-well plates, and 10^4^ viral copies of SARS-CoV-2 of each strain were inoculated into each well in triplicates. The viruses were infected using a dilution ratio of 10-fold dilution. After adsorption for 1 h at 37 °C in a humidified atmosphere with 5% CO_2_, the medium was removed, infected cells were washed with DMEM/5%FBS, and fresh medium (DMEM containing 5% FBS) was added. After 72 h, cells were observed to evaluate the cytopathic effect. Then, 10 µL of Cell Counting Kit-8 (CCK-8) (Dojindo Molecular Technologies, Kumamoto, Japan) was added into each well and incubated for 1 h at 37 °C. Then, the cell viability was analyzed at the 450 OD. An improved Spearman–Kärber method was used to calculate the results, which are presented as TCID_50_/mL [47].

### 4.7. Gene Expression Assay Using Quantitative Real-Time PCR

Post-infection Calu-3 cell total RNA was extracted using the RNeasy Mini Kit (QIAGEN, Tokyo, Japan) following the manufacturer’s instructions. The RNA quality was assessed with the Agilent High-Sensitivity RNA ScreenTape System (Agilent Technologies, Inc., Waldbronn, Germany). cDNA was synthesized using the Superscript^TM^ IV VILO^TM^ Master Mix with the ezDNAase Enzyme Kit (Thermo Fisher, Tokyo, Japan). Gene expression was measured on the QuantStudio 5 system (Thermo Fisher Scientific, Waltham, MA, USA) with the TaqMan^TM^ Gene Expression Assay method. Predesigned primers for gene expression analysis are detailed in Table 4. We included three biological replicates to study the gene expression profiles for each time point: 0, 12, 24, and 48 h post-infection (hpi). The housekeeping gene ATF4, which does not exhibit significant differential expression during SARS-CoV-2 infection, was used as the reference gene for RT-qPCR. Gene expression levels are presented as fold changes relative to the untreated controls using the 2−∆∆CT method. All assays were performed in triplicate, including a no-template control (NTC).

### 4.8. Next-Generation Sequencing

The SARS-CoV-2 genome sequencing protocol utilized the QIAseq FX DNA Library Kit (QIAGEN, GmbH, Hilden, Germany) with an Illumina sequencing platform. Viral RNA was reverse-transcribed using the LunaScript RT SuperMix Kit (NewEngland Biolabs, Ipswich, MA, USA). Multiplex PCR amplification was performed using the Q5 Hot Start High-Fidelity DNA Polymerase (New England Biolabs, Ipswich, MA, USA) and NIID ver. N5 primer sets [48]. Purification of PCR products was conducted using Agencourt AMPure XP beads (Beckman Coulter, Brea, CA, USA), followed by pooling in low-TE buffer (10 mM Tris-HCl, pH 8.0, 0.1 mM EDTA). The library preparation involved fragmentation, end-prep, and adapter ligation with reagents provided in the Qiagen kit, incorporating indexed adapters for sample identification. Libraries were pooled, purified, and quantified with a Qubit Flex Fluorometer (Invitrogen, Thermo Fisher Scientific, Waltham, MA, USA). Denatured and diluted libraries were analyzed by following the Miseq protocol. The prepared libraries were loaded into Miseq Reagent Kit v2 (Illumina, San Diego, CA, USA) and sequenced on an Illumina platform, adhering to strict contamination control measures and including negative controls to ensure quality. Bioinformatics analyses were subsequently performed for genome assembly and variant identification.

### 4.9. Genomic Sequencing Analysis

We analyzed genomic sequence data by aligning paired-end reads to the reference genome (EPI_ISL_402124.fasta) using BWA v0.7.17. The aligned reads were processed into sorted and indexed BAM files to facilitate variant calling. Single-nucleotide polymorphisms (SNPs) and insertions/deletions (indels) were identified using GATK v4.6.0 with the HaplotypeCaller tool. To ensure data robustness, variables were filtered using quality control thresholds, including QD < 2.0, QUAL < 30.0, and MQ < 40 for SNPs, with analogous criteria for indels. The filtered variant datasets were annotated and compiled in VCF, TSV, and Excel formats, enabling comprehensive downstream analysis and visualization.

### 4.10. RNA Sequencing

Total RNA was extracted using an RNeasy Mini Kit (QIAGEN, Tokyo, Japan) and subjected to quality assessment using the TapeStation system (Agilent Technologies, Santa Clara, CA, USA). Samples meeting the criteria of an RNA quantity of ≥3 μg, a concentration of ≥65 ng/μL, an OD260/280 ratio of ≥1.8, and an RNA integrity number (RIN) of ≥7.0 were selected for sequencing. mRNA was enriched via poly-A selection, and the sequencing libraries were constructed using the TruSeq Stranded mRNA Library Prep Kit (Illumina, San Diego, CA, USA), yielding an average insert size of 300–600 bp. Paired-end sequencing (2 × 100 bp) was performed on the Illumina NovaSeq 6000 platform (Illumina, San Diego, CA, USA), and it generated approximately 4 Gb of data (~40 million reads) per sample in FASTQ format.

### 4.11. RNA-Sequencing Analysis

To ensure the integrity of our RNA-sequencing data, we removed adapter sequences and trimmed low-quality bases using Cutadapt v4.1. Adapter sequences were identified with a minimum overlap of 10 bases, and bases with a Phred quality score below 20 were trimmed. Reads shorter than 51 bases were excluded to maintain the reliability of downstream analyses. The processed RNA-seq reads were aligned to the reference genome (GCF_015252025.1_Vero_WHO_p1.0) and its corresponding gene annotation file using RSEM v1.3.3, with STAR being employed as the aligner. We accounted for strand-specific library preparation during alignment and generated BAM-format files for mapped reads. Quantification of gene expression levels was conducted, followed by differential expression analysis using edgeR v3.28.1. Differentially expressed genes (DEGs) were identified using an FDR of <0.05. Heatmaps and clustering analyses of significant genes were created using Heatmaply v1.3.0, and the results were visualized. The analysis was performed by GeneBay, Inc. (Yokohama, Japan).

### 4.12. Gene Ontology (GO) Enrichment Analysis

A GO enrichment analysis was conducted to identify biological processes, cellular components, and molecular functions associated with differentially expressed genes (DEGs). The workflow involved three main steps: DEG identification and visualization, GO enrichment analysis, and result interpretation. The first step involved identifying differentially expressed genes (DEGs) based on the following statistical thresholds: false discovery rate (FDR) ≤ 0.05 and log2 fold change (|log2FC| ≥ 1). Additionally, heatmaps and clustering analysis were performed using the heatmaply package (version 1.3.0), which normalized expression data using log10-transformed TPM values. These visualizations helped in identifying patterns and similarities across different sample groups. Following DEG identification, GO enrichment analysis was performed using clusterProfiler (version 3.14.3).

The reference Gene Ontology annotation file (GCF_015252025.1_Vero_WHO_p1.0_gene_ontology.gaf) was used to map genes to GO terms. The analysis was conducted separately for each experimental group to ensure the identification of functionally relevant patterns. *p*-Values were adjusted using the Benjamini–Hochberg (BH) method to control false discovery rates. The results were categorized into three main functional groups: biological processes (BPs), cellular components (CCs), and molecular functions (MFs). The results of the analysis were stored in structured output files. The tabular results contained enriched GO terms along with *p*-values, *q*-values, gene counts, and gene ratios. Additionally, bar plots were generated to illustrate the number of genes associated with each GO term and their statistical significance.

### 4.13. Statistical Analysis

The 2−∆∆CT method was used for the statistical analysis of quantitative reverse-transcription PCR (RT-qPCR) data [49]. The Shapiro–Wilk test for normality was utilized to confirm that there was a normal distribution before conducting parametric analysis. A two-tailed *t*-test was used to statistically analyze the viral copy numbers, growth kinetics, and TCID_50_. Statistical analysis was performed with GraphPad Prism software (version 10.0). *p*-values of ≤0.05 are indicated with a single asterisk (*), (**) *p* < 0.01, (***) *p* < 0.001, and (****) *p* < 0.0001.

### 4.14. Generative AI Disclosure

The author(s) verify and take full responsibility for the use of generative AI in the preparation of this manuscript. ChatGPT-4o (OpenAI, Version: March 2025) was used for language assistance, with all content having been thoroughly reviewed by the authors.

## 5. Conclusions

This study provides a detailed analysis of the viral characteristics of and host response to persistent SARS-CoV-2 isolates from clinical samples. The findings highlight the significant changes in viral infectivity and underscore the virus’s adaptive capabilities within the host. Genomic mutation might affect the gene expression analysis, revealing substantial alterations in host response mechanisms and emphasizing the roles of key receptors, antiviral genes, and cytokines in managing SARS-CoV-2 infection. Future studies are pivotal to exploring the genetic changes in the persistent virus over time and their implications for viral fitness, transmissibility, and pathogenicity. Understanding these dynamics is crucial for developing targeted therapies to manage persistent SARS-CoV-2 infections and mitigate long-term complications.

## Figures and Tables

**Table 1:** Patients’ clinical data.

Patient ID	Age/Gender	Duration of Infection	Comorbidities	Symptoms	Vaccination Status	Immuno-Suppressive Medication	Complication	ICU (Yes/No)	Treatment Drug (D/R/M) *	Outcome
Patient 1	79/M	57 Days	Esophageal cancer, rheumatoid arthritis, pneumonia	Fever	No	-	Pneumonia. ARDS	No	D, R (total 12 days)	Discharged (transferred to previous hospital for rehab)
Patient 2	37/F	37 Days	Pregnancy	Fever, cough, nausea	Vaccinated 2x	-	-	No	No	Discharged
Patient 3	66/F	46 Days	Lung transplant, pneumonia, CKD, Bowen’s disease	Fever, sore throat	No	Prednisolone, mycophenolate mofetil	pneumonia	No	M (total 5 days)	Discharged
Patient 4	76/M	58 Days	Lung cancer, hypertension	Fever	Unknown	-	-	No	R (total 5 days)	Discharged

* Treatment drug; D: dexamethasone; R: remdesivir; M: molnupiravir.

**Table 2:** List of changes in single-nucleotide polymorphisms (SNPs) between early-phase and late-phase infections.

No.	Gene Region	Protein	Nucleotide		Type	Change	
			Start	End		Reference	Alternate
1	ORF1ab	Nsp2	465	465	SNP	C	T
2		Nsp2	515	518	DEL	GTTA	G
3		Nsp3	2485	2485	SNP	C	T
4		Nsp12	10,401	10,401	SNP	C	T
5		Nsp13	10,936	10,936	SNP	T	G
6		Nsp13	11,414	11,414	SNP	C	T
7		Nsp13	11,750	11,750	SNP	C	T
8		Nsp14	15,526	15,526	SNP	A	G
9		Nsp15	16,178	16,178	SNP	C	T
10		Nsp16	17,762	17,762	SNP	C	T
11		Nsp16	17,766	17,766	SNP	C	T
12		Nsp16	17,977	17,977	SNP	C	T
13		Nsp16	19,380	19,380	SNP	C	T
14		Nsp16	19,824	19,824	SNP	G	A
15	S	Spike	21,986	21,995	DEL	GGTGTTTATT	G
16		Spike	21,987	21,987	SNP	G	A
17		Spike	22,498	22,498	SNP	C	T
18		Spike	23,804	23,804	SNP	G	A
19		Spike	24,062	24,062	SNP	A	T
20		Spike	24,452	24,452	SNP	A	G
21	ORF3a	Ap3a	25,677	25,677	SNP	G	T
22		Ap3a	25,998	25,998	SNP	A	G
23	ORF7a	Ap7a	27,505	27,510	DEL	GGAACA	G
24		Ap7a	27,524	27,524	SNP	C	T
25	ORF7b	Ap7b	27,794	27,797	DEL	TTTA	T
26	ORF8	Ap8	28,077	28,077	SNP	G	T
27	N	Nucleoprotein	28,539	28,539	SNP	G	T

**Table 3:** List of primers for quantitative real-time PCR (qPCR).

Name	Sequence (5′-3′)
2019 nCoV N F2 primer	AAATTTTGGGGACCAGGAAC
2019 nCoV N R2 primer	TGGCAGCTGTGTAGGTCAAC
2019 nCoV N P2 probe	FAM-ATGTCGCGCATTGGCATGGA-QSY

**Table 4:** List of primers/probes for real-time RT-PCR (TaqManTM Gene Expression Assay, Applied Biosystems).

Gene Expression	Primer Sequence Number	Base Pairs
ATF4	Hs00909569_g1	68
TMPRSS2	Hs01122322_m1	62
ACE2	Hs01085333_m1	141
DDX58	Hs01061436_m1	65
IFNB1	Hs01077958_s1	73
IFNAR2	Hs01022059_m1	88
IL6	Hs00174131_m1	95
IL1B	Hs01555410_m1	91
TNF	Hs00174128_m1	80
ULBP2	Hs01127964_m1	93

## Data Availability

All data generated or analyzed during this study are included in this published article.
